# Phosphoproteomics Reveals HMGA1, a CK2 Substrate, as a Drug-Resistant Target in Non-Small Cell Lung Cancer

**DOI:** 10.1038/srep44021

**Published:** 2017-03-14

**Authors:** Yi-Ting Wang, Szu-Hua Pan, Chia-Feng Tsai, Ting-Chun Kuo, Yuan-Ling Hsu, Hsin-Yung Yen, Wai-Kok Choong, Hsin-Yi Wu, Yen-Chen Liao, Tse-Ming Hong, Ting-Yi Sung, Pan-Chyr Yang, Yu-Ju Chen

**Affiliations:** 1 Chemical Biology and Molecular Biophysics Program, Taiwan International Graduate Program, Institute of Chemistry, Academia Sinica, Taipei, 11529, Taiwan; 2 Institute of Biochemical Sciences, National Taiwan University, Taipei, 10617, Taiwan; 3 Graduate Institute of Medical Genomics and Proteomics, College of Medicine, National Taiwan University, Taipei, 10617, Taiwan; 4 Genome and Systems Biology Degree Program, National Taiwan University and Academia Sinica, Taipei, 11529, Taiwan; 5 Ph. D. Program in Translational Medicine, National Taiwan University and Academia Sinica, Taipei, 11529, Taiwan; 6 Institute of Chemistry, Academia Sinica, Taipei, 11529, Taiwan; 7 Department of Chemistry, National Taiwan University, Taipei, 10617, Taiwan; 8 Genomics Research Center, Academia Sinica, Taipei, 11529, Taiwan; 9 Institute of Information Science, Academia Sinica, Taipei, 11529, Taiwan; 10 Graduate Institute of Clinical Medicine, College of Medicine, National Cheng Kung University, Tainan, 70101, Taiwan; 11 Department of Internal Medicine, National Taiwan University Hospital and National Taiwan University Medical College, Taipei, 10617, Taiwan; 12 Institute of Biomedical Science, Academia Sincia, Taipei, 11529, Taiwan

## Abstract

Although EGFR tyrosine kinase inhibitors (TKIs) have demonstrated good efficacy in non-small-cell lung cancer (NSCLC) patients harboring EGFR mutations, most patients develop intrinsic and acquired resistance. We quantitatively profiled the phosphoproteome and proteome of drug-sensitive and drug-resistant NSCLC cells under gefitinib treatment. The construction of a dose-dependent responsive kinase-substrate network of 1548 phosphoproteins and 3834 proteins revealed CK2-centric modules as the dominant core network for the potential gefitinib resistance-associated proteins. CK2 knockdown decreased cell survival in gefitinib-resistant NSCLCs. Using motif analysis to identify the CK2 core sub-network, we verified that elevated phosphorylation level of a CK2 substrate, HMGA1 was a critical node contributing to EGFR-TKI resistance in NSCLC cell. Both HMGA1 knockdown or mutation of the CK2 phosphorylation site, S102, of HMGA1 reinforced the efficacy of gefitinib in resistant NSCLC cells through reactivation of the downstream signaling of EGFR. Our results delineate the TKI resistance-associated kinase-substrate network, suggesting a potential therapeutic strategy for overcoming TKI-induced resistance in NSCLC.

Lung cancer is the leading cause of cancer-related deaths worldwide. According to the World Health Organization (WHO), non-small cell lung carcinoma (NSCLC) is the predominant type of lung cancer, constituting approximately 75–80% of all lung cancers. While advances in treatment have been made in the last 20 years, the response rate of lung patients to chemotherapy is less than 30%, and patient prognosis remains poor^1^. To address this situation, the development of new anticancer drugs that target specific molecular pathways activated in cancers, such as epidermal growth factor receptor (EGFR) signaling, has brought excitement in lung cancer studies in the middle of the last decade^2^. Inhibition of EGFR kinase activity using antibodies directed against the extracellular domain of EGFR (such as cetuximab) or small molecules that specifically inhibit the tyrosine kinase activity of EGFR (such as gefitinib) have emerged as alternative treatments for patients with lung cancer. The EGFR tyrosine kinase inhibitors (EGFR-TKIs) are now widely adapted as therapeutic strategy owing to their higher response rate in patients with EGFR mutations such as exon 19 deletion and L858R^3^.

Although the response rate to EGFR-TKIs is approximately 80% in NSCLC patients harboring an EGFR mutation, progression-free survival is less than 1 year, as most patients develop intrinsic and acquired resistance to EGFR-TKIs^4^. This situation stimulated interest in understanding how TKI resistance develops. Although mechanisms such as an acquired secondary mutation of the EGFR gene at threonine 790 (T790M, 50%) and c-Met amplification (20%)^5^ have been reported to be correlated with acquired resistance, the mechanisms accounting for the remaining 30% of drug-resistant patients are still unclear, and further study is required to identify new therapeutic targets for the effective treatment of EGFR-TKI resistance^6^.

Abnormal protein kinase activities and the corresponding changes in the protein phosphorylation state have been implicated in the onset of tumor formation and cancer progression^7^; and therefore become attractive targets for the development of therapeutic agents to treat cancer as well as drug resistance^8^,^9^. However, the direct identification of kinases or kinase-substrate pairs remains a major barrier for understanding cell signaling networks. Sequence motif analysis^10^ has provided clues to map the corresponding kinases. *Dephoure et al.* identified two unique motifs derived from thousands of phosphopeptides, suggesting the existence of two undiscovered kinases related to cell mitosis^11^. Based on kinase-motif analysis using a linear motif atlas^12^, reported that three kinases (ATM, ATR, and DNA-dependent protein kinases) were highly activated during mitotic S phase of the DNA damage response network. Imami, *et al*.^13^ described the temporal response of phosphorylation dynamics of the kinase inhibitor lapatinib. Through motif analysis and *in vitro* and *in vivo* kinase profiling, PKA was identified as the putative kinase mediating HER2 serine/threonine phosphorylation. The above studies demonstrated that phosphoproteomics and subsequent motif-based analysis might effectively allow the proteome-wide profiling of a signaling network and the identification of kinase-substrate pairs.

To identify the altered phosphorylation events associated with dose-dependent responsiveness and drug resistance, we performed label-free quantitative phosphoproteomics in drug-sensitive PC9 cells and drug-resistant PC9/gef cells following gefitinib treatment. Mapping the kinase-substrate network associated with drug resistance may facilitate the identification of better drug targets. Based on the hypothesis that a drug-resistant target might be up-regulated in drug-resistant cells but would show no response upon further gefitinib treatment, we categorized the trend of phosphorylation changes matched to different kinase motif to facilitate target selection. We further constructed a protein-protein interaction network of the dominant kinase and performed motif analysis to identify their corresponding substrates associated with gefitinib resistance. Here, we present the interesting finding that CK2 and HMGA1 might be involved in EGFR-TKI resistance, as supported by biochemical and cell biology experiments. These results may provide new insight to define a critical signaling node associated with the development of EGFR-TKI resistance for NSCLC treatment in the future.

## Results

**Figure 1:**
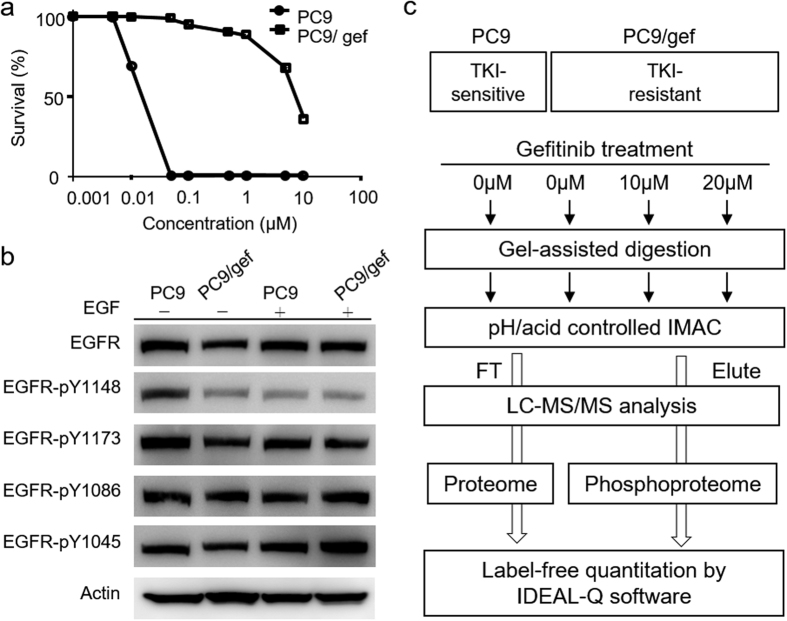
TKI response of PC9 and PC9/gef cell lines and EGFR phosphorylation profile. (**a**) Cell survival rates of PC9 and PC9/gef cells under treatment with various concentrations of gefitinib. (**b**) Phosphorylation profile of EGFR kinase active sites with or without EGF stimulation. Site-specific antibodies against pY1148, pY1173, pY1086 and pY1045 were used for western blot detection. The intensity of phosphorylation sites were normalized to the intensity of total EGFR protein intensity in comparison analysis. (**c**) Experimental workflow of the quantitative proteomic and phosphoproteomic analysis of TKI-sensitive PC9 and TKI-resistant PC9/gef cells under gefitinib treatment (10 μM and 20 μM). The label-free quantitation approach integrated gel-assisted digestion and pH/acid-controlled IMAC for phosphopeptide purification. After IMAC purification, the eluted fraction was used for phosphoproteome analysis, while the flow-through fraction was used for protein-level quantitation. The quantitation of protein and phosphopeptides was performed using Ideal-Q software.

To obtain a global view of the aberrant phosphoproteomic profiles associated with EGFR-TKI-induced drug resistance in NSCLC, we performed quantitative phosphoproteomics in a pair of TKI-sensitive (PC9) and TKI-resistant (PC9/gef) cell lines. PC9 is a gefitinib-sensitive cell line harboring an EGFR exon 19 deletion, and its derivative PC9/gef is a resistant cell line that was selected from parental PC9 cells after continuous exposure to an increasing concentrations of gefitinib^14^,^15^. First, we confirmed the TKI resistance of these two cell lines through a sulforhodamine B (SRB) assay and determined the IC_50_ values for gefitinib. As shown in Fig. 1a, the calculated IC_50_ values were 0.02 μM and 7.75 μM for PC9 cells and PC9/gef cells, respectively. EGFR activity was evaluated using western blotting to detect the phosphorylation status of these two cell lines. An EGFR-activating mutation would result in autophosphorylation of the kinase domain, whereas wild-type EGFR would form a dimer, and the phosphorylation of its kinase domain would increase upon EGF activation^16^. According to the *PhosphoSitePlus*^®^ database, pY1086 has multiple functions, including playing roles in cell motility^17^ and internalization^18^. Phosphorylation at pY1045 prevents EGFR degradation^19^. Phosphorylation of pY1148 and pY1173 is required for kinase enzymatic activity^20^. As shown in Fig. 1b, all four sites of EGFR (pY1148, pY1173, pY1086, and pY1045) remained consistently phosphorylated either with or without EGF treatment in both cell lines, consistent with the reported autophosphorylation in the kinase domain of mutated EGFR. When PC9 and PC9/gef cells were compared, pY1086, and pY1045 show only slightly increased phosphorylation signal in PC9/gef cells. However, the expression level of pY1148 showed a lower basal level in PC9/gef cells, which became even lower upon EGF treatment in PC9/gef cells, suggesting that the kinase activity of EGFR pY1148 may be lower in the resistant cells. Taken together, these results suggest that EGFR signaling partially contributed to the difference between gefitinib-sensitive PC9 and resistant PC9/gef cells and hint at the existence of an alternative activated signaling pathway, which may play an essential role in the development of TKI resistance in NSCLC cells.

Next, we aimed to determine the alternative phosphorylation signaling networks associated with TKI resistance in NSCLC. We performed quantitative phosphoproteomics to compare the TKI-sensitive PC9 and TKI-resistant PC9/gef cells upon gefitinib treatment. Compared with PC9 cells, we hypothesized that the drug-resistant target might show an overly activated phosphorylation status in resistant PC9/gef cells to drive drug resistance. Therefore, the dose-dependent effects were also explored in PC9/gef cells under adjunctive high-dose treatment (10 μM or 20 μM) with gefitinib. As shown in the experimental workflow (Fig. 1c), the eluent and flow-through fraction of immobilized metal affinity chromatography (IMAC) were used for quantitation of the phosphoproteome and proteome, respectively.

**Figure 2:**
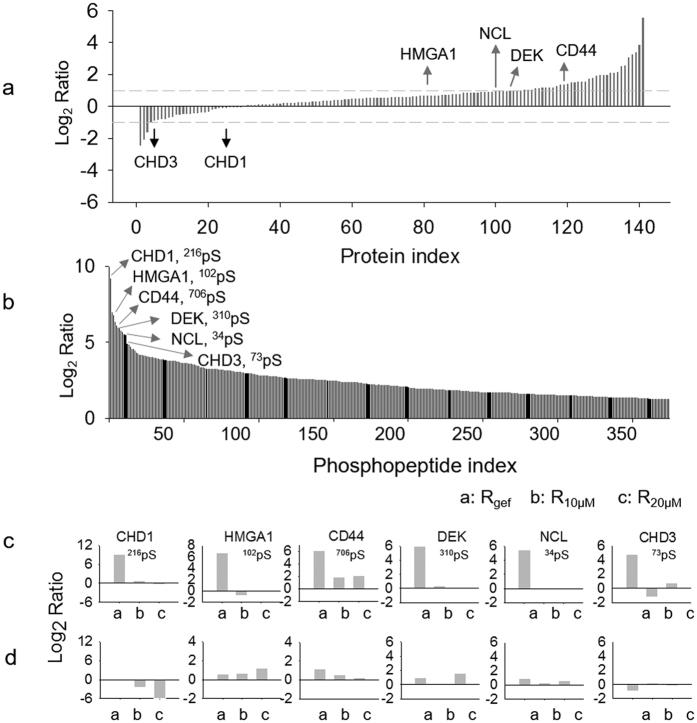
Summary of quantitative analysis of the phosphoproteome and proteome. (**a**) The histogram shows the quantitation ratios (R_gef_, i.e., PC9/gef cells compared with PC9 cells) of the protein expression levels of 141 proteins exhibiting up-regulated phosphopeptide levels in PC9/gef cells. (**b**) The histogram indicated the quantitation ratios of 375 up-regulated phosphopeptides in R_gef_. The top six phosphoproteins with the highest up-regulated ratios were selected as examples to demonstrate the changes in phosphorylation levels (**c**) and protein levels (**d**).

The quantitative phosphoproteomic analysis identified 5844 unique phosphorylation sites from 4612 phosphopeptides in 1160 proteins. A total of 3835 proteins were identified in the flow-through fraction. Between these two datasets, 651 proteins overlapped, indicating that 17.6% of the total identified proteins could be quantified in terms of phosphorylation and protein levels. As shown in Supplementary Fig. S1a and b, quantitative comparisons under three conditions were plotted with a normal distribution: (1) PC9/gef compared with PC9 (hereafter, ratio: R_gef_) and treatment with (2) 10 μM or (3) 20 μM gefitinib in PC9/gef cells compared with PC9/gef cells (hereafter, ratio: R_10μM_ and R_20μM_, respectively). The pie chart shown in Supplementary Fig. S1c and d indicates the number of phosphopeptides that were up-regulated, down-regulated or unchanged in R_gef_, R_10μM_ and R_20μM_. The detailed quantitation results for the phosphoproteome and proteome are listed in Supplementary Tables S1 and S2, respectively. The expression levels of 66~81% of the quantified proteins were unchanged, while as many as 50% of the phosphopeptides showed alterations, suggesting that the changes in phosphorylation were more dramatic than the changes at the protein level. Using the R_gef_ group as an example (Fig. 2a), among the 458 up-regulated (>2-fold) phosphopeptides identified, protein level ratios were available in our proteome dataset for 375 phosphopeptides corresponding to 141 proteins. Among these 141 proteins, most (99 proteins) did not show any difference in their levels between the PC9 and PC9/gef cells. In Fig. 2b, this trend is exemplified for the top 6 phosphopeptides exhibiting the greatest changes in the R_gef_ group. Compared to PC9 cells, all these 6 phosphopeptides showed an increased intensity (log_2_ ratio of 2.5–9.2) in resistant PC9/gef cells (Fig. 2c, Bar “a”). Interestingly, 5 of them did not show significant change in the protein expression (Fig. 2d, DEK, R_gef_ = 0.95; NCL, R_gef_ = 0.89; HMGA1, R_gef_ = 0.55; CHD1, R_gef_ = −0.08; and CHD3, R_gef_ = −0.91), while only CD44 exhibited a slightly up-regulated protein level in the R_gef_ group (R_gef_ = 1.21) (Fig. 2d). However, under a high dose of gefitinib in PC9/gef cells (R_10μM_ and R_20μM_ groups), the protein and phosphopeptide levels of most of the proteins remained similar, revealing that gefitinib treatment has a limited ability to either activate or suppress these six proteins.

**Figure 3:**
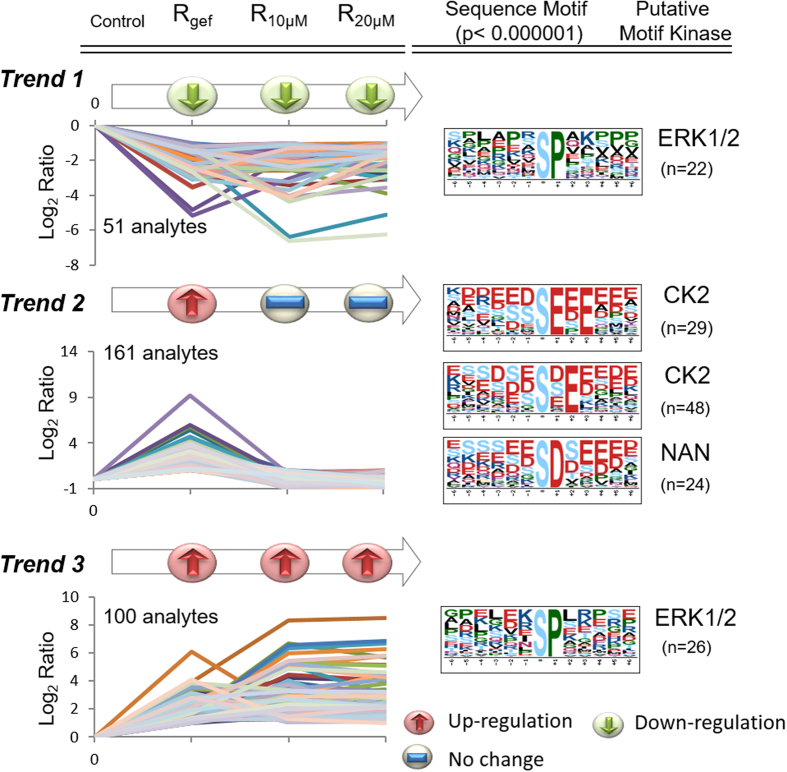
The phosphopeptides showing different levels under gefitinib treatment were categorized according to three trends. The red arrow, green arrow and blue line represent up-regulation, down-regulation and no change, respectively. Under *Trend 1*, phosphopeptides showing 2-fold down-regulation in R_gef_, R_10μM_ and R_20μM_ were selected. ERK1/2 sequence motifs (S-P) were enriched from 22 phosphopeptides in the *Trend 1* group with a score of 9.94. Under *Trend 2*, phosphopeptides exhibiting a greater than 2-fold up-regulation of phosphorylation in R_gef_, but no change in R_10μM_ and R_20μM_, were selected. Two CK2 sequence motifs, (S-E-X-E) and (S-X-E), were enriched from 29 and 48 phosphopeptides in the *Trend 2* group with scores of 30.56 and 16, respectively. The sequence motif (S-D) was matched with 24 phosphopeptides with a score of 16, but no putative kinases were matched. Under *Trend 3*, phosphopeptides displaying greater than 2-fold up-regulation in R_gef_, R_10μM_ and R_20μM_ were selected. ERK1/2 sequence motifs (S-P) were enriched from 26 phosphopeptides in the *Trend 3* group with a score of 11.04.

The responsiveness to treatment under a high dose of gefitinib may be reflected by the differential expression ratios determined for R_gef,_ R_10μM_ and R_20μM._ To assess potential drug-resistant targets, the phosphopeptides were grouped according to three trends, *Trend 1* (gefitinib-inhibited targets), *Trend 2* (gefitinib-resistant targets), and *Trend 3* (gefitinib-activated targets), based on the ratios obtained for R_gef_, R_10μM_ and R_20μM_ (Fig. 3). The quantitation results for these 3 trends are listed in Supplementary Tables S3,S4 and S5. During the transition of PC9 to PC9/gef cells, which is obtained by culturing with a low dose of gefitinib, the activity of gefitinib-responsive kinases is expected to be decreased by the long-term treatment of gefitinib. This hypothesis can be verified by the 14 phospho-serine motifs extracted from 1109 down-regulated phosphopeptides in R_gef_ (Supplementary Table S6). Among these kinases, ERK1/2, PKA, PKC, CDK and GSK-3 have been reported to be downstream of EGFR pathways. The decreased phosphorylation levels of their substrates were consistent with findings that EGFR kinase activity decreased, while the phosphorylation of sites (pY1086 and pY1045) responsible for EGFR degradation slightly increased (Fig. 1b). Through further treatment with a high dose of gefitinib, these drug-sensitive targets may be continuously suppressed. Thus, the phosphopeptides that were continuously down-regulated in R_gef,_ R_10μM_ and R_20μM_ under *Trend 1* might represent drug inhibition targets. These results also suggest that EGFR pathways might not be dominant in PC9/gef cells due to their drug resistance and that other overexpressed proteins or kinases might support cell survival.

However, under our hypothesis, the higher phosphorylation status of the targets in R_gef_ (i.e., constitutively activated phosphorylation in resistant PC9/gef) may potentially drive drug resistance. Upon further gefitinib treatment of PC9/gef cells, these potential drug-resistant targets may lose their ability to respond to higher doses of gefitinib treatment. Thus, the second criterion for selecting resistant targets was based on unchanged R_10μM_ and R_20μM_ ratios, which indicated no response to gefitinib. As shown in Fig. 3, *Trend 2* represents our hypothetical drug-resistant targets, exhibiting up-regulation in R_gef_ and no changes in R_10μM_ and R_20μM_. Under *Trend 3*, the phosphopeptides that showed up-regulation of the three ratios may represent constitutively gefitinib-activated targets.

To identify the key kinase responsible for regulating the drug resistance mechanism, we extracted phosphorylation motifs from the phosphopeptides in each trend group using Motif-X and searched for their corresponding putative kinases in the Human Protein Reference Database (HPRD). As shown in Fig. 3, ERK1/2 (S-P) was enriched under *Trend 1* (p < 0.000001) and *Trend 3* (p < 0.000001). Based on the 161 phosphopeptides that passed the filtering criteria in the *Trend 2* group, CK2 was enriched as a major kinase with 2 substrate motifs, (S-X-E) and (S-E-X-E). In addition, the protein expression level of CK2 was higher in PC9/gef cells rather than PC9 cells (R_gef_: 2.4-fold) and remain unchanged in R_10μM_ (0.9-fold) and R_20μM_ (0.8-fold) (Supplementary Table S2). It is noted that CK2 was also identified as the major kinase in the dominant core phosphoprotein network with higher basal level of phosphorylation stoichiometry in resistant NSCLC cells^21^. Therefore, CK2 may be an attractive candidate for developing a novel therapeutic strategy for EGFR-TKI resistance lung cancer.

**Figure 4:**
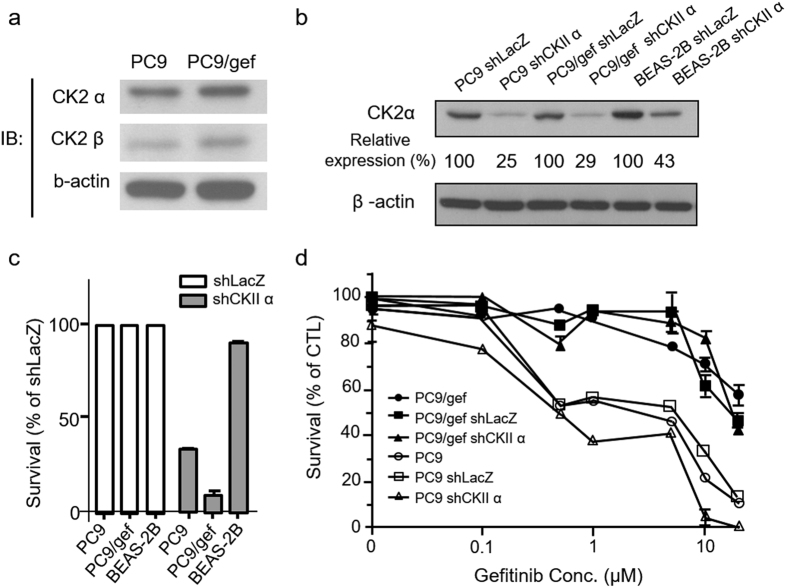
CK2 knockdown induces cell apoptosis yet does not regulate EGFR-TKI resistance in PC9 and PC9/gef cells. (**a**) Western blot analysis of CK2 alpha and beta subunit expression in PC9 and PC9/gef cells. (**b**) Western blot analysis of the efficiency of CK2α knockdown in PC9, PC9/gef and BEAS-2B cells. (**c**) Histogram of the cell survival rate (%) after knockdown using control shRNA, shLacZ and shCK2α (mean ± SE in triplicate experiments). (**d**) Cytotoxicity assays for various concentrations of gefitinib in CK2α-knockdown PC9 and PC9/gef cells.

CK2 is a serine/threonine kinase composed of two alpha and two beta subunits, where the alpha subunits contain the catalytic kinase domain^22^. First, the protein expression levels of CK2α and CK2β were validated by western blotting. As shown in Fig. 4a, the expression levels of CK2α and CK2β were higher in PC9/gef cells than that in PC9 cells; these results are consistent with mass spectrometry (MS)-based quantitation results. Next, we knocked down the expressions of CK2 to study its role in PC9 and PC9/gef cells. The efficiency of CK2 knockdown was confirmed by the reduced relative expression of CK2 by 25%, 29% and 43% in PC9, PC9/gef and BEAS-2B cells (a normal bronchial epithelium cell line used as a negative control), respectively (Fig. 4b). Knockdown of CK2 had pronounced effects on the proliferation of PC9/gef cells; the percentage of survival was less than 9% in CK2-deficient PC9/gef cells compared with the shLacZ control of PC9/gef cells (Fig. 4c). In comparison, CK2 knockdown had a much weaker effect on the survival of PC9 cells (34% reduction of the survival percentage compared with PC9/shLacZ) and showed no effect on the non-cancerous bronchial epithelium BEAS-2B cells (91% compared with the shLacZ control of BEAS-2B). The results indicate that CK2 is essential for the survival of NCSLC cells.

To evaluate whether knocking down CK2 expression can enhance the sensitivity of gefitinib in both PC9 and PC9/gef cells, we measured the IC_50_ of gefitinib in the remained cells after knockdown of CK2. As shown in Fig. 4d, knockdown of CK2 only slightly decreased the IC_50_ of gefitinib in the remaining PC9 cells (34%) and did not have an effect on the remaining surviving PC9/gef cells (9%). Collectively, these findings indicate that CK2 may play a critical role in cell survival in both PC9 and PC9/gef cells and could be a good therapeutic target for reducing tumor growth. However, the results showing that knockdown of CK2 in PC9/gef cells did not enhance the sensitivity of gefitinib exclude a potential role of CK2 in recovery from EGFR-TKI resistance.

**Figure 5:**
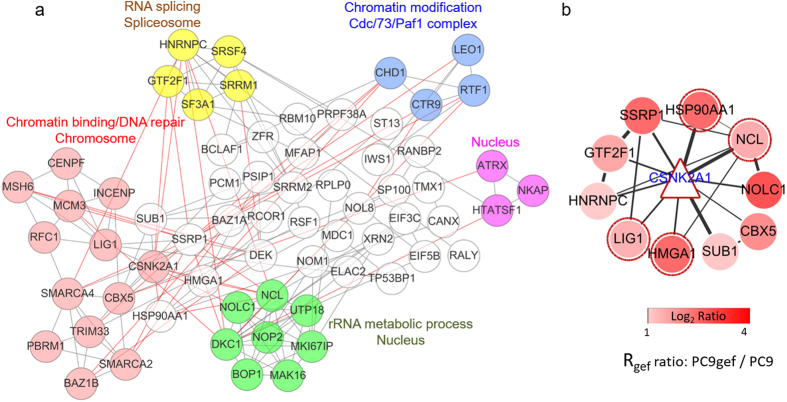
Network analysis of differentially expressed phosphoproteins in the *Trend 2* group constructed using String and Cytoscape software. CSNK2A1, also known as CK2, was added to analyze CK2-interacting phosphoproteins. (**a**) Further functional enrichment with DAVID online software revealed clusters of proteins connected to various biological functions and cellular components. (**b**) The first-neighborhood protein-protein interactions of CK2 were selected. Within the CK2 network, four proteins, HSP90AA1, NCL, LIG1 and HMGA1, have been reported to play roles in lung cancer and are marked with red circles. The various red color in the filled circle indicates the different ratios of the selected phosphorylation sites in PC9/gef cells and PC9 cells.

CK2 regulates a large number of critical signaling networks; thus, identification of its downstream substrate-dependent pathways may allow a more specific effect on the regulation of EGFR-TKI resistance to be achieved. Therefore, we further dissected the potential downstream substrates of CK2 as potential targets responsible for EGFR-TKI resistance. The phosphorylation of most of the protein substrates by their kinases occurs through protein-protein interactions (PPI). We therefore constructed a CK2-centered (gene symbol: CSNK2A1) PPI network of the 82 phosphoproteins in the *Trend 2* group through String, Cytoscape and Gene Ontology analysis. The results showed that 65 of the 82 phosphoproteins from *Trend 2* were connected to CK2 kinase through a previously annotated PPI in the constructed network (Fig. 5a). The analysis also revealed clusters of proteins connected to various biological functions and cellular components. Among the five clusters, the two largest networks were connected to two different functions: “DNA repair” and “rRNA metabolic processes”. Both functions are related to chemotherapy, and some of these nodes have been reported to be cancer biomarkers. Burger *et al*. observed that inhibition of ribosome biogenesis, including rRNA metabolic processes, by the chemotherapeutic drugs flavopiridol and 5-fluorouracil could potentially increase the efficacy of therapeutic treatment in human fibrosarcoma^23^. In addition, chemotherapeutic drugs induce DNA damage, which is a mechanism that allows cells to repair damage and confer resistance to anticancer drugs^24^. In the “rRNA metabolic process” module, NCL and NOLC1 have been reported as prognostic and diagnostic markers in lung cancer^25^; UTP18 can promote tumorigenesis in many human cancers, and the correlation between UTP18 overexpression and decreased survival of neuroblastoma and breast cancer patients suggests its potential utility as a prognostic marker^26^. In the “DNA repair” module, most of the protein nodes have been reported to relate to cancer. For example, CBX5 is overexpressed in lung cancer and can promote cell survival^27^, and driver mutations of PBRM1, a tumor suppressor gene, cause protein inactivation and tumor growth in renal cell carcinomas^28^. Other modules such as “Chromatin modification” and “RNA splicing”, consists of components of ribosome biogenesis that help prevent DNA damage and might therefore also be related to chemotherapy in NSCLC. Furthermore, in addition to CK2, the network facilitated the identification of HMGA1, SSRP1 and HSP90AA1, which may be responsible for the crosstalk between the “DNA repair” and “rRNA metabolic process” categories.

To investigate aberrant phosphoproteins that are potentially related to drug resistance, we further focused on the first-layer CK2-centric PPI network, which included 9 proteins with a CK2 kinase motif, HMGA1, LIG1, GTF2F1, HSP90AA1, HNRNPC, NCL, SSRP1, CBX5, SUB1, and NOLC1, located in the first-neighbor protein interaction network of CK2 (Fig. 5b). Among the nine proteins, LIG1, NCL, HSP90AA1, and HMGA1 have been reported to be related to lung cancer. Inherited variants of LIG1 were associated with predisposition to smoking-related lung cancer^29^. Due to its ability to act as a molecular chaperone that can stabilize many onco-proteins, HSP90 has been reported as a druggable target in many cancers, including ALK-rearranged NSCLC, HER2-amplified breast cancer and some hematological malignancies (e.g., multiple myeloma). However, inhibition of HSP90 simultaneously down-regulates several redundant pathways that are crucial for cell viability and might cause side effects during treatment^30^. NCL overexpression is inversely correlated with the survival rate in lung cancer^25^. HMGA overexpression is a feature of most neoplastic tissues, including lung cancer^31^. Despite their roles related to lung cancer, the kinases associated with the identified phosphorylation sites matching the CK2 phosphorylation motif, including serine 141 of LIG1, serines 263 and 252 of HSP90AA1 and serines 206, 28 and 34 of NCL, have not been reported.

**Figure 6:**
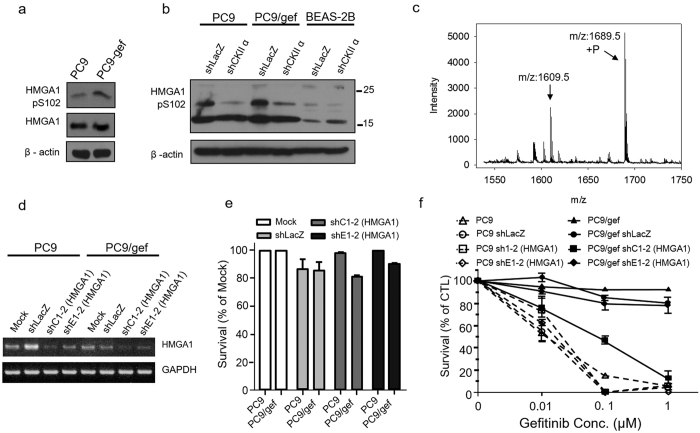
HMGA1 regulates drug resistance in NSCLC. (**a**) Validation of HMGA1 through western blotting using an HMGA1-pS102 site-specific antibody and an HMGA1 antibody. (**b**) Knockdown of CK2 reduces HMGA1 phosphorylation. (**b**) Confirmation that HMGA1 is a CK2 substrate through an *in vitro* kinase assay using synthetic peptides of HMGA1 and CK2 kinase, followed by detection via MALDI-TOF MS. (**d**) RT-PCR analysis of the efficiency of HMGA1 knockdown in PC9 and PC9/gef cells; shLacZ virus infection was used as a negative control. (**e**) Histogram of the cell survival rate (%) after knockdown using control shRNA, shLacZ and shHMGA1 (C1-2 and E1-2) (mean ± SE in triplicate experiments). (**f**) Cytotoxicity assays for various concentrations of gefitinib in HMGA1-knockdown PC9 and PC9/gef cells.

Among these four phosphoproteins, only serine 102 from HMGA1 matching the CK2 kinase motif (S-X-X-E) has been demonstrated to be phosphorylated via a CK2 kinase reaction^32^. Thus, we selected the HMGA1 protein for further verification due to its differential expression, kinase-substrate relationship and potential function in regulating gefitinib-induced resistance in PC9/gef cells. As shown in Fig. 6a, western blotting analysis using a phospho-site-specific anti-pSer102 HMGA1 antibody supported the finding that pSer102 of HMGA1 showed higher levels in PC9/gef cells than in PC9 cells, while the protein expression levels of HMGA1 did not change in the two cell lines. These results indicate that the elevated phosphorylation of serine 102 was not due to protein overexpression (Fig. 6a). As a control, our results also indicated that phosphorylated HMGA1 displayed a lower expression level in BEAS-2B cells than in PC9 and PC9/gef cells (Fig. 6b), suggesting that HMGA1 phosphorylation was overexpressed in NSCLC. After knockdown of CK2, the Ser102 phosphorylation levels of HMGA1 in PC9, PC9/gef and BEAS-2B cells vanished (Fig. 6b), confirming that HMGA1 is a substrate of CK2 kinase. The kinase-substrate relationship was further confirmed using an *in vitro* kinase assay in which a synthetic HMGA1 peptide (93-107 AA) was allowed to react with the CK2 kinase, followed by matrix-assisted laser desorption/ionization-time of flight ((MALDI-TOF) mass spectrometry detection. As illustrated in Fig. 6c, after the CK2 kinase reaction, the synthetic peptide (m/z: 1609.5) exhibited an 80-Da mass shift to an m/z value of 1689.5 Da, which represented the signal of its phosphorylated form. In contrast, when the reaction was carried out with MAPK1 kinase as a negative control, no mass shift of the synthetic HMGA1 peptide occurred (Supplementary Fig. S2). These results demonstrate that HMGA1 is a substrate of the CK2 kinase.

We then explored the function of HMGA1 in lung cancer by knocking down its expression in PC9 and PC9/gef cells. Following shRNA virus infection with the shC1-2 clone, the expression level of HMGA1 mRNA was significantly knocked down by nearly 70% in both PC9 and PC9/gef cells (Fig. 6d). Next, we chose these stable cell lines to examine whether the reduced expression of HMGA1 may be correlated with the response to gefitinib in both PC9 and PC9/gef cells. The results regarding cell viability indicated that silencing the expression of HMGA1 did not affect the growth of PC9 and PC9/gef cells (Fig. 6e). Instant cell death was observed in HMGA1-deficient PC9/gef cells (shC1-2, Fig. 6f) under treatment with only 1 μM gefitinib. Although the efficiency of HMGA1 knockdown was not 100%, the IC_50_ was greatly reduced, from near 10 μM in PC9/gef cells to 0.1 μM in HMGA1-deficient PC9/gef cells. As expected, HMGA1 knockdown had a profound effect on recovering the sensitivity of the response to gefitinib in the initially resistant PC9/gef cells. To evaluate the role of identified phosphorylation site S102 on HMGA1 to enhance the sensitivity of gefitinib in PC9/gef cells, we performed the defective mutation on Ser102 of HMGA1. Similar result of reduced IC_50_ was obtained from the cells that were transfected with the mutated construct, pCIneo-HMGA1 S102 (Supplementary Fig. 3). The results suggest that HMGA1 is a potential gefitinib-resistant target in TKI-resistant NSCLC cells and that knockdown of HMGA1 could turn these TKI-resistant NSCLC cells into TKI-sensitive cells.

**Figure 7:**
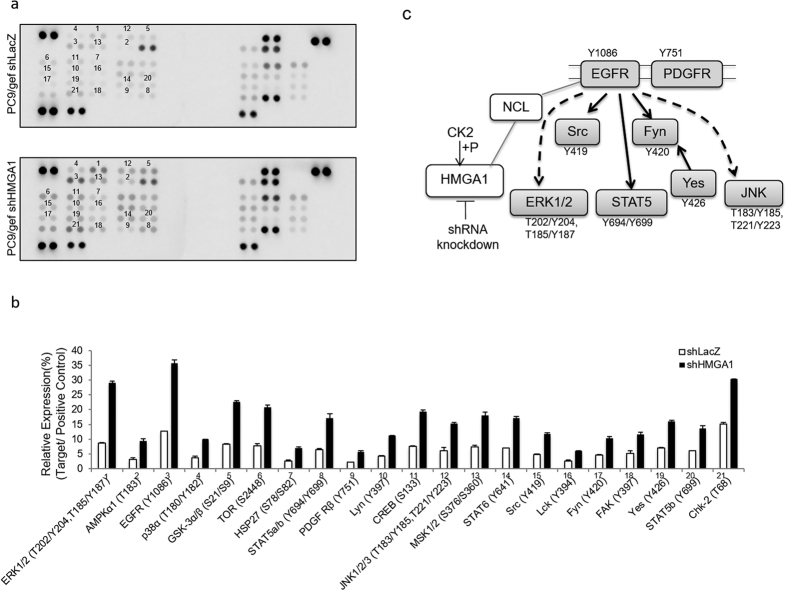
HMGA1 knockdown influences EGFR pathways. (**a**) Phopsho-kinase profiles in PC9/gef shLacZ and PC9/gef shC1-2 (HMGA1) obtained by human phospho-kinase array (R&D Systems, ARY003). Each membrane contains kinase specific (for example, 1−21) and positive control (P) antibodies spotted in duplicate. (**b**) Relative phosphorylation level was quantified by normalizing the pixel density of each positive control and shown in percentage. (**c**) Model of the mechanism of HMGA1-knockdown to recover sensitivity to gefitinib in resistant NSCLC.

To understand how silencing of HMGA1 is involved in recovering the sensitivity of genifitinib in lung cancer cells, we utilized phospho-kinase array to investigate which signaling pathways were activated after knocking down the expression of HMGA1 in PC9/gef cells. The results showed that 21 protein kinases, including ERK1/2 (T202/Y204, T185/Y187), AMPKα1 (T183), EGFR (Y1086), p38α (T180/Y182), GSK-3α/β (S21/S9), TOR (S2448), HSP27 (S78/S82), STAT5a/b (Y694/Y699), PDGF Rβ (Y751), Lyn (Y397), CREB (S133), JNK1/2/3 (T183/Y185, T221/Y223), MSK1/2 (S376/S360), STAT6 (Y641), Src (Y419), Lck (Y394), Fyn (Y420), FAK (Y397), Yes (Y426), STAT5b (Y699) and Chk-2 (T68) exhibited different degree of increased phosphorylation level in PC9/gef shHMGA1 cells compared with PC9/gef shLacZ control cells (Fig. 7a and b). Further pathway analysis by DAVID software revealed that some of these protein kinases are related to the EGFR signaling modules including ERK 1/2, Src, STAT5, Fyn, Yes and JNK or PDGFR (Fig. 7c). This implies that the reversed sensitivity of gefitinib by silencing HMGA1 may be due to the re-activation of the EGFR- or PDGFR- downstream signaling in NSCLC cells. Through incorporation the phosphoproteomics dataset, we further identified that NCL protein, which has 5-fold (Log_2_ ratio) increased phosphorylation level at serine 34 site (Fig. 2c), may act as a critical hub between EGFR, HMGA1 and CK2. The NCL had been reported to have protein-protein interaction with EGFR on the cytosolic tail^33^, HMGA1 in the adhesion complex^34^ and CK2 in human autophagy system^35^. Whether NCL is likely to be the mediator between HMGA1 and the EGFR signaling remain further study (Fig. 7c). Nevertheless, all these data suggest HMGA1 may play as a critical hub in gefitinib resistance in NSCLC.

## Discussion

Abnormal protein kinase activities and the corresponding changes in downstream phosphorylation-mediated signaling have been implicated in the onset of tumor formation and cancer progression and have therefore become attractive targets for therapeutic agents for the treatment of cancer and drug resistance^36^. To discover drug-resistant targets, global genomic approaches have been the conventional methods used to identify key genes and pathways related to the mechanism of drug resistance, enabling the rational design of new anticancer drugs to overcome drug resistance^37^. However, these methods do not provide information on protein posttranslational modifications, such as phosphorylation, that could lead to the identification of abnormal kinases driving drug resistance. In recent years, MS-based quantitative proteomic analysis has made it possible to utilize large-scale phosphoproteomic profiles for the discovery of drug-resistant targets. Based on a tyrosine phosphoproteomic analysis, Gioia *et al*. observed increased phosphorylation of Lyn and Syk kinase in nilotinib-resistant chronic myeloid leukemia (CML) cells. They further confirmed that co-expression of Lyn and Syk was required to fully induce resistance to nilotinib in drug-sensitive CML cells and that inhibition of Syk restored the capacity of nilotinib to inhibit cell proliferation^38^. By constructing tamoxifen-perturbed signaling pathways using phosphoproteomic analysis, Browne, *et al*.^39^ discovered a substrate protein, the myristoylated alanine-rich C-kinase substrate (MARCKS) protein, as a potential biomarker for anti-estrogen tamoxifen-resistant breast cancer. However, functional study by knockdown of MARCKS did not show effect regarding the reversal of drug resistance. These studies revealed the promise of phosphoproteomic approaches for the effective identification of abnormal protein kinases and corresponding substrate phosphoproteins that are involved in drug resistance in cancer.

In the present study, the phosphoproteomic profiling of gefitinib-sensitive and gefitinib-resistant lung cancer cell lines led to the establishment of a database of drug resistance-associated proteins in NSCLC. Within the potentially targetable phosphoproteomic network, we identified elevated site-specific phosphorylation of CK2 and its substrate HMGA1 as being associated with gefitinib resistance. Further functional analysis revealed that CK2 decreases cell survival in drug-resistant NSCLC, but no effect regarding recovery from gefitinib resistance was observed. CK2 is overexpressed in many cancers, including hematologic malignancies such as chronic lymphocytic leukemia (CLL)^40^, acute myeloid leukemia (AML)^41^, T-cell acute lymphocytic leukemia (T-ALL)^42^ and multiple myeloma^43^. CX-4945, also known as Silmitasertib, is a highly specific, ATP-competitive inhibitor of CK2 that induces cytotoxicity and apoptosis by suppressing the activation of the CK2-mediated PI3K/Akt/mTOR signaling pathways and is currently being evaluated in clinical trials for the treatment of many types of cancer, including hematological malignancies and bile duct cancers^44^. Prins *et al*. reported that the CK2 inhibitor CX-4945 could enhance the efficacy of the chemotherapy drug fludarabine in primary CLL cells through inhibition of the BCR pathway^45^. Bliesath, *et al*.^46^ found that CK2 and EGFR signaling could cooperate to promote oncogenic signaling in NSCLC, and they further demonstrated that the synergistic combination of a CK2 inhibitor with EGFR antagonists reduced tumor size in a murine NSCLC xenograft. These studies have focused on the utility of CK2 as an anti-cancer target and demonstrated that combinatorial use of CX-4945 is a promising therapeutic tool for the treatment of cancer; however, its relationship with drug resistance has not been determined.

The HMGA proteins are small, low-molecular-weight (thus high mobility group) proteins with an AT-hook DNA-binding domain^47^. In this study, HMGA1, an activated substrate of CK2, was demonstrated to be a potential drug-resistant target for the recovery of TKI sensitivity in NSCLC. High expression of HMGA1 has been observed in neoplastic tissues^31^, including the pancreas^48^, colon^49^, breasts^50^, and lungs. Emerging evidences have demonstrated that HMGA1 is a promising therapeutic target for multiple cancer types. Liau *et al*.^51^ reported that HMGA1 silencing could increase apoptosis activity and reduce the IC_50_ of gemcitabine to enhance chemosensitivity in pancreatic cells. The function of HMGA1 in regulating metastatic progression has been described in colon and breast cancers. Belton, *et al*.^52^ found that HMGA1 controlled proliferative changes and polyposis formation in the intestines of transgenic mice and induced metastatic progression and stem-like properties in colon cancer cells, suggesting that HMGA1 could be a rational therapeutic target in metastatic colon cancer. Shah, *et al*.^53^ also reported the regulatory role of HMGA1 in relation to stem cell properties in triple-negative (resistant) breast cancer cells. These authors observed that silencing HMGA1 could block oncogenic properties, including proliferation, migration, invasion, and tumorigenesis, in triple-negative (resistant) breast cancer cells by reprogramming cancer cells through stem cell transcriptional networks.

The current understanding of the role of HMGA1 in lung cancer is limited. In NSCLC, Zhang, *et al*.^54^ discovered that HMGA1 binds directly to the proximal promoter of miR-222 and regulates oncogenetic miR-222 transcriptional activity. Hillion *et al*.^55^ revealed that inhibition of HMGA1 expression could decrease cell growth in metastatic large-cell carcinoma lung cancer cells. Here, we identified HMGA1 within the over activated CK2-substrate network by mapping the differential phosphoproteomic profiles between TKI-sensitive and resistant NSCLC cells under dose-dependent TKI treatment. Further analysis indicated that knockdown of HMGA1 expressions could reinforce gefitinib efficacy in resistant PC9 cells, and this effect may be due to the re-activation of EGFR or PDGF downstream signaling. Moreover, through incorporation the phosphoproteomics dataset, we first identified that NCL protein may play as a critical hub between EGFR, HMGA1 and CK2 via protein-protein interaction relationship network. These four proteins had been reported as overexpressed proteins in lung cancer, but how these molecules connect with each other and have end results in EGFR-TKI resistance should be further clarified. Our results provide the first line of evidence indicating HMGA1 as a potential drug-resistant target in gefitinib-induced resistant NSCLC.

In conclusion, phosphoproteomic identification and kinase–substrate motif analysis allowed us to link kinases with intracellular signaling networks, leading to new perspectives regarding kinases and their substrates as targets relate to drug resistance in NSCLC. Further studies are necessary to delineate the molecular function of HMGA1 in resistant NSCLC and to explore rational combination therapy and its underlying mechanism.

## Experimental Procedures

### Agents and antibodies

RPMI 1640 medium, fetal bovine serum (FBS), penicillin, streptomycin, and all other cell culture reagents were obtained from GIBCO/BRL Life Technologies (Grand Island, NY). Antibodies to CK2α, CK2β, HMG-I/HMG-Y, anti-mouse and anti-rabbit IgGs were obtained from Santa Cruz Biotechnology, Inc (Santa Cruz, CA, USA). Antibody to β-actin was obtained from Sigma–Aldrich (St. Louis, MO). Sulforhodamine B (SRB), trichloroacetic acid (TCA), Dimethyl sulfoxide (DMSO), Hexadimethrine bromide (polybrene) Triethylammonium bicarbonate (TEABC), iron-(III) chloride (FeCl3), formic acid, acetic acid, HPLC-grade acetonitrile (ACN) and all of the other chemical reagents were obtained from Sigma-Aldrich (St Louis, MO, USA). Lipofectamine 2000 and Opti-MEM were obtained from Life Technologies (Carlsbad, CA, USA). A stock solution of gefitinib, a gift from AstraZeneca (London, UK), was prepared in dimethyl sulfoxide and stored at −20 °C. The BCA protein assay kit was obtained from Pierce (Rockford, IL, USA). Modified sequencing-grade trypsin was purchased from Promega (Madison, WI, USA). Ammonium persulfate (APS) and N,N,N′,N′-tetramethylenediamine (TEMED) were purchased from Amersham Pharmacia (Piscataway, NJ, USA). Polypropylene frits disk was purchased from Agilent (Wilmington, DE, USA). Ni-NTA silica resin was purchased from Qiagen (Hilden, Germany). SDB-XC Empore disks were obtained from 3 M (St. Paul, MN). Water was obtained from a Millipore Milli-Q system (Bedford, MA).

### Cell lines and cell culture

The human lung adenocarcinoma cell line PC9 and derivative PC9/gef clones were gifts from Dr. C. H. Yang (Graduate Institute of Oncology, Cancer Research Center, National Taiwan University). The PC9 cells were cultured in 30 nM gefitinib in the beginning. For every subculture, the dead cells were discard and the remaining survival cells were cultured in the does of 10 nM more gefitinib concentration. The final concentration of gefitinib was 5 μM. The normal human bronchial epithelium cell lines BEAS-2B, and the human embryonic kidney cell lines HEK293 and HEK293T (transformed using sheared HAd5 DNA to render it sensitive to human adenovirus and permissive to adenovirus DNA) were purchased from American Type Culture Collection (Rockville, MD, USA). PC9, PC9/gef, and BEAS-2B were cultured in RPMI-1640 medium with 10% FBS (v/v) and penicillin (100 units/mL)/streptomycin (100 μg/mL). HEK293 and HEK293T were cultured in Dulbecco’s modified Eagle’s medium with 10% FBS (v/v) and penicillin (100 units/mL)/streptomycin (100 μg/mL). Cultures were maintained in a humidified incubator at 37 °C in 5% CO2/95% air.

### Western Blotting

Cancer cells from each cancer line were harvested, washed three times with PBS, and lysed in lysis buffer (0.25 M Tris-HCl, pH 6.8, 0.1% SDS). The protein concentration was measured by BCA assay. Then, the protein samples were separated on 4–12% NuPAGE (Invitrogen) and transferred to PVDF membranes (Millipore). The membranes was blocked with blocking buffer (5% skim milk in TBS) for 1 hr, and then incubated with anti-EGFR, anti-EGFR phosphosite-specific antibodies and anti-HMGA1 antibody (all from cell signaling), anti-pSer102 HMGA1 antibody (GeneTex) or anti-CK2 antibody (SANTA CRUZ) by 1:1000 diluted in blocking buffer. After washing with TBST (0.05% Tween-20 in TBS), the membranes were incubated with peroxidase-conjugated second antibodies for developing the signal.

### Gel-assisted Digestion

The protein samples from NSCLC cell lines were subjected to gel-assisted digestion^56^. By using acrylamide/bisacrylamide solution (40%, v/v, 29:1), 10% (w/v) ammonium persulfate, 100% N, N, N′, N′-tetramethylenediamine and protein samples by a 5:0.7:0.3:14ratio (v/v), the protein sample was fixed into a gel directly in the Eppendorf. The gel containing protein samples was cut into small gel pieces and washed 3 times with 25 mM TEABC containing 50% (v/v) ACN followed by dehydrating with 100% ACN and completely drying by vacuum centrifugation. Then, protein samples were digested by Trypsin (protein:trypsin = 50:1, g/g) in 25 mM TEABC at 37 °C overnight. The extraction of tryptic peptides were performed 3 times with 5% (v/v) FA in 50% (v/v) ACN for 30 min and dried completely by vacuum centrifugation at room temperature.

### IMAC Procedure

Phosphopeptides enrichment were carried out by using an IMAC protocol^57^,^58^. The in-house-made IMAC tip was capped in a tip-end with a 20 μm polypropylene frits disk followed by packing with 20 mg of Ni-NTA silica resin. Firstly, Ni^2+^ ions were replaced with Fe^3+^ by washing with 50 mM EDTA in 1 M NaCl and activated with 100 mM FeCl_3_. Secondly, tryptic peptides were reconstituted in 6% (v/v) AA and loaded onto the IMAC tip and the flow-through (FT) was collected. Thirdly, IMAC tip was washed by 6% (v/v) AA, 25% ACN, and followed by 6% (v/v) AA. Finally, the bound peptides were eluted with 200 mM NH_4_H_2_PO_4_. The flow-through and eluted peptides fractions were desalted using reversed phase-StageTips (SDB-XC).

### LC-MS/MS Analysis

Purified phosphopeptides and flow-through peptides were reconstituted in buffer A (0.1% FA in H_2_O) (v/v) and performed triplicate LC-MS/MS analysis. NanoLC−nanoESI-MS/MS analysis was performed on a nanoAcquity system (Waters, Milford, MA) connected to an LTQ-Orbitrap Velos mass spectrometer (Thermo Fisher Scientific, Bremen, Germany) equipped with a nanospray interface (Proxeon, Odense, Denmark). Peptide mixtures were loaded onto a 75 μm ID, 25 cm length C18 BEH column (Waters, Milford, MA) packed with 130 Å pore size and 1.7 μm particles and were separated in 30 min from 5% to 40% gradient solvent B (ACN with 0.1% FA) at a flow rate of 300 nl/min and a column temperature of 35 °C. The data-dependant mode of mass spectrometer was operated with full scan MS spectra acquired in the orbitrap (*m/z* 350–1600, resolution set to 60,000 at *m/z* 400 and automatic gain control (AGC) target at 10^6^) and 10 most intense ions were isolated for CID MS/MS fragmentation and detected in linear ion trap (AGC target at 7000, dynamically excluded for 90 sec) sequentially.

### Database Search

RAW2MSM (version 1.1.) software was used to perform raw MS/MS data format transformation to msm-files for peptide sequence search. Mascot search engine against the Swisssprot Homo_sapiens database were performed with the following parameters were allowed: tryptic peptides with 0 to 2 missed cleavage sites; the parent ion tolerance was 10 ppm and the fragment ion mass tolerance was 0.6 Da; specifically for phosphopeptide search, phosphorylation (STY) and oxidation(M) were set as variable modifications; whereas protein search, only oxidation(M) was set as variable modifications. Mascot Significance threshold for peptide identification is set as p < 0.05. All of the mass spectrometry derived proteomics and phosphoproteomics datasets have been submitted in the ProteomeXchange Consortium (http://proteomecentral.proteomexchange.org) via the PRIDE partner repository with the dataset identifier PXD000375 and DOI 10.6019/PXD000375.

### Quantitative Analysis by IDEAL-Q

The quantitation of phosphoproteomics and proteomics were performed by the SEMI label free algorithm by using IDEAL-Q software^59^,^60^. Firstly, ReAdW (XCalibur, Thermo Finnigan) program was used to convert the raw data files acquired from the LTQ-Orbitrap into mzXML file format for peptide peak reconstruction. Secondly, the search results in MASCOT were exported in eXtensive Markup Language data (.XML) file format for merging to a global peptide information list (sequence, elution time and mass-to-charge). Thirdly, peptide information list was used for elution time alignment with linear regression in different LC-MS/MS runs and followed by correction the aberrant chromatographic shift across segment time domains. To increase correct assignment confidence, the detected peptide peaks would meet the criteria: (a) accurate charge state and (b) correct isotope pattern (c) signal-to-noise (S/N) ratio >3. Finally, the IDEAL-Q software reconstructed extracted ion chromatography (XIC), and integrated the XIC area for relative peptide abundance calculation. The fold-change of a given phosphopeptide and protein would be calculated between different samples. The protein ratio was determined by a weighted average of the peptide ratios, where the weight of each peptide ratio is determined by the sample abundance of the corresponding peptide.

### Lentivirus production and transduction

The LacZ- and CK2α-, HMGA1(C1-2)-, HMGA1(D1-2)-, HMGA1(E1-2)-shRNA containing lentiviral vectors were obtained from the National RNAi Core Facility (Academia Sinica, Taipei, Taiwan) and prepared in accordance with standard protocols. In brief, HEK293T cells were co-transfected with the indicated lentiviral vector and two helper plasmids, pCMVΔR8.91 and pMD.G, by using Lipofectamine 2000 reagents according to manufacturer’s protocols. Virus-containing medium was collected at 24-, 48- and 72-h post-transfection. To knockdown the indicated genes in the cells, cells were infected with lentivirus in medium containing polybrene (8 μg/ml). After twenty-four hours post-infection, cells were treated with fresh medium for 24–48 hours and then used for all experiments.

### RNA extraction and reverse transcription polymerase chain reaction (RT-PCR)

Total RNAs were extracted by TRIzol (Invitrogen) and 1 μg total RNA was used in cDNA synthesis with random hexamer primers using Superscript III reverse transcriptase (Invitrogen). HMGA1 gene was amplified with the following pairs of primers: 5′-ATGAGTGAGTCGAGCTCGAA-3′ (sense) and 5′-TCACTGCTCCTCCTCCGA-3′ (antisense) and GAPDH gene was amplified with the following pairs of primers: 5′-GAAGGTGAAGGTCGGAGTC-3′ (sense) and 5′-GAAGATGGTGATGGGATTTC-3′ (antisense). After denaturation at 95 C for 5 min, PCR was performed with PCR master mix reagent (GMbiolab) for 30 cycles in HMGA1 amplification and 22 cycles in GAPDH amplification. Each reaction cycle includes denaturation at 95 C for 30 sec, annealing at 55 C for 30 sec, and extension at 72 °C for 30 sec, followed by a final extension at 72 C for 10 min. PCR products were analyzed on 2% agarose gel in TBE running buffer (Sigma–Aldrich), and visualized in the presence of 1 mg/ml ethidium bromide staining.

### Plasmid Constructs and Transfection

The cDNAs encoding full-length human HMGA1 was amplified from the CL (human lung adenocarcinoma) cell line by polymerase chain reaction (PCR). The amplified cDNAs were subcloned into the pCIneo (Clontech, Mountain View, CA) vectors for generation of full-length HMGA1 plasmid construct. Then, the mutated plasmid, pCIneo-HMGA1 S102, was generated via PCR-directed mutagenesis according to the manufacturer’s instructions (QuickChange kit; Stratagene).

For expression the full-length or pSer102 mutated recombinant proteins, plasmids pCIneo-HMGA1 and pCIneo-HMGA1 S102 were transfected into 70% confluent PC9/gef cells using Lipofectamine 3000 reagents according to the manufacturer’s protocol. Thirty-six hours after transfection, cells were prepared for performing Sulforhodamine B (SRB) assay to determine their IC_50_ of gefitinib and western blotting for examination of their protein expressions.

### Sulforhodamine B (SRB) assay

2 × 10^3^ cells were cultured in 96-well culture plates for 24 h before use in the experiment. The culture medium was replaced with fresh medium containing the appropriate concentration of compound ranging from 0.005 μM to 10 μM for 72 h. After an incubation period, the cells were fixed with 10% trichloroacetic acid and stained for 30 min, after which the excess dye was removed by washing repeatedly with 1% acetic acid. The protein-bound dye was dissolved in 10 mM Tris base solution for OD determination at 510 nm using a microplate reader. The cell growth curve was plotted using GraphPad software.

### *In vitro* kinase reaction

5 μL of 0.11 mM HMGA1 (93–107 AA) (1 μg) were mixed with 5 μL of 2X kinase reaction buffer from Promega ADP Glo kinase assay (160 mM Tris–HCl, 80 mM MgCl_2_, 0.2 mM DTT), 5 μL of 250 μM ATP solution (Promega), and 4 μL of kinases as kinase (New England Biolabs Inc.). The mixture was reacted under constant shaking at 30 °C for 4 hours. The reaction was stopped by acidifying the solution with TFA in 0.5% v/v final concentration on ice. The mixture was desalted and followed by IMAC procedure for phosphopeptides enrichment as previously described.

### MALDI-TOF MS Analysis

Phosphopeptide from *in vitro* kinase assay was performed by 4800 MALDI TOF/TOF Analyzer (Applied Biosystems, Foster City, CA, USA). 0.5 μL of enriched phosphopeptides was mixed with 0.5 μL of matrix (20 mg/mL 2,5-dihydroxybenzoic acid (DHB) in 50% ACN and 1% H3PO4). MS was performed by positive reflector mode with the setting of 20 kV accelerated voltage, 16% grid voltage, and low-mass gate of 1000 Da. One spectrum was composed by 1200 laser pulses. Data-Explorer software (Applied Biosystems) was used for raw spectra processing of baseline subtraction and noise removement.

### Human Phospho-Kinase Profiles Analysis

The kinase phosphorylation profiles were analyzed by the Human Phospho-Kinase Array (R&D Systems) following the protocol provided by the manufacturer. In brief, 10^7^ PC9/gef shLacZ and shHMGA1 cells were solubilized in 1 mL lysis buffer and clarified by centrifugation of 12000 rpm at 4 °C for 30 min. The supernatant were collected as total cell lysates, diluted with the array buffer that provided by the commercial kit and incubated with the membrane with shaking at 4 °C overnight. Then, the membranes were incubated with the antibody cocktail and the streptavidin-HRP signals were detected. The exposure images were further quantified by ImageJ software (NIH, Bethesda, MD); pixel density was evaluated and calculated.

## Additional Information

**How to cite this article:** Wang, Y.-T. *et al*. Phosphoproteomics Reveals HMGA1, a CK2 Substrate, as a Drug-Resistant Target in Non-Small Cell Lung Cancer. *Sci. Rep.* **7**, 44021; doi: 10.1038/srep44021 (2017).

**Publisher's note:** Springer Nature remains neutral with regard to jurisdictional claims in published maps and institutional affiliations.

## Supplementary Material

Supplementary Information

Supplementary Dataset 1

## References

[b1] Nguyen K. S. & Neal J. W. First-line treatment of EGFR-mutant non-small-cell lung cancer: the role of erlotinib and other tyrosine kinase inhibitors. Biologics. 6, 337–345, doi: 10.2147/BTT.S26558 (2012). 23055691 PMC3459550

[b2] Mitsudomi T. Advances in target therapy for lung cancer. Jpn. J. Clin. Oncol. 40, 101–106, doi: 10.1093/jjco/hyp174 (2010). 20031962

[b3] da Cunha Santos G., Shepherd F. A.& Tsao M. S. EGFR mutations and lung cancer. Annu. Rev. Pathol. 6, 49–69, doi: 10.1146/annurev-pathol-011110-130206 (2011). 20887192

[b4] Laurent-Puig P., Lievre A. & Blons H. Mutations and response to epidermal growth factor receptor inhibitors. Clin. Cancer Res. 15, 1133–1139, doi: 10.1158/1078-0432.CCR-08-0905 (2009). 19228718

[b5] Guix M. . Acquired resistance to EGFR tyrosine kinase inhibitors in cancer cells is mediated by loss of IGF-binding proteins. J. Clin. Invest. 118, 2609–2619, doi: 10.1172/JCI34588 (2008). 18568074 PMC2430495

[b6] Kosaka T., Yamaki E., Mogi A. & Kuwano H. Mechanisms of resistance to EGFR TKIs and development of a new generation of drugs in non-small-cell lung cancer. J. Biomed. Biotechnol. 2011, 165214, doi: 10.1155/2011/165214 (2011). 21687596 PMC3114474

[b7] Sathornsumetee S. & Reardon D. A. Targeting multiple kinases in glioblastoma multiforme. Expert Opin. Investig. Drugs 18, 277–292, doi: 10.1517/13543780802692603 (2009). 19243279

[b8] Giamas G., Stebbing J., Vorgias C. E. & Knippschild U. Protein kinases as targets for cancer treatment. Pharmacogenomics 8, 1005–1016, doi: 10.2217/14622416.8.8.1005 (2007). 17716234

[b9] Ralph S. J. An update on malignant melanoma vaccine research: insights into mechanisms for improving the design and potency of melanoma therapeutic vaccines. Am. J. Clin. Dermatol. 8, 123–141 (2007). 17492842 10.2165/00128071-200708030-00001

[b10] Schwartz D. & Gygi S. P. An iterative statistical approach to the identification of protein phosphorylation motifs from large-scale data sets. Nat. Biotechnol. 23, 1391–1398, doi: 10.1038/nbt1146 (2005). 16273072

[b11] Dephoure N. . A quantitative atlas of mitotic phosphorylation. Proc. Natl. Acad. Sci. USA 105, 10762–10767, doi: 10.1073/pnas.0805139105 (2008). 18669648 PMC2504835

[b12] Miller M. L. . Linear motif atlas for phosphorylation-dependent signaling. *Sci*. Signal. 1, ra2, doi: 10.1126/scisignal.1159433 (2008). PMC6215708 18765831

[b13] Imami K. . Temporal profiling of lapatinib-suppressed phosphorylation signals in EGFR/HER2 pathways. Mol. Cell. Proteomics 11, 1741–1757, doi: 10.1074/mcp.M112.019919 (2012). 22964224 PMC3518135

[b14] Chang T. H. . Slug confers resistance to the epidermal growth factor receptor tyrosine kinase inhibitor. Am. J. Respir. Crit. Care Med. 183, 1071–1079, doi: 10.1164/rccm.201009-1440OC (2011). 21037017

[b15] Koizumi F., Shimoyama T., Taguchi F., Saijo N. & Nishio K. Establishment of a human non-small cell lung cancer cell line resistant to gefitinib. Int. J. Cancer Suppl. 116, 36–44, doi: 10.1002/ijc.20985 (2005). 15761868

[b16] Gazdar A. F. Activating and resistance mutations of EGFR in non-small-cell lung cancer: role in clinical response to EGFR tyrosine kinase inhibitors. Oncogene 28 Suppl 1, S24–31, doi: 10.1038/onc.2009.198 (2009). 19680293 PMC2849651

[b17] Cardoso A. P. . Macrophages stimulate gastric and colorectal cancer invasion through EGFR Y(1086), c-Src, Erk1/2 and Akt phosphorylation and smallGTPase activity. Oncogene 33, 2123–2133, doi: 10.1038/onc.2013.154 (2014). 23644655

[b18] Jiang X., Huang F., Marusyk A. & Sorkin A. Grb2 regulates internalization of EGF receptors through clathrin-coated pits. Mol. Biol. Cell 14, 858–870, doi: 10.1091/mbc.E02-08-0532 (2003). 12631709 PMC151565

[b19] Ahsan A. . Role of epidermal growth factor receptor degradation in cisplatin-induced cytotoxicity in head and neck cancer. Cancer Res. 70, 2862–2869, doi: 10.1158/0008-5472.CAN-09-4294 (2010). 20215522 PMC2848889

[b20] Sorkin A., Helin K., Waters C. M., Carpenter G. & Beguinot L. Multiple autophosphorylation sites of the epidermal growth factor receptor are essential for receptor kinase activity and internalization. Contrasting significance of tyrosine 992 in the native and truncated receptors. J. Biol. Chem. 267, 8672–8678 (1992). 1314835

[b21] Tsai C. F. . Large-scale determination of absolute phosphorylation stoichiometries in human cells by motif-targeting quantitative proteomics. Nat. Commun. 6, 6622, doi: 10.1038/ncomms7622 (2015). 25814448 PMC4389224

[b22] Montenarh M. Protein kinase CK2 and angiogenesis. Adv. Clin. Exp. Med. 23, 153–158 (2014). 24913104 10.17219/acem/37040

[b23] Burger K. & Eick D. Functional ribosome biogenesis is a prerequisite for p53 destabilization: impact of chemotherapy on nucleolar functions and RNA metabolism. Biol. Chem. 394, 1133–1143, doi: 10.1515/hsz-2013-0153 (2013). 23640940

[b24] Holohan C., Van Schaeybroeck S., Longley D. B. & Johnston P. G. Cancer drug resistance: an evolving paradigm. Nat. Rev. Cancer 13, 714–726, doi: 10.1038/nrc3599 (2013). 24060863

[b25] Zhao H. . Prognostic significance of the combined score of endothelial expression of nucleolin and CD31 in surgically resected non-small cell lung cancer. PLoS One 8, e54674, doi: 10.1371/journal.pone.0054674 (2013). 23382938 PMC3561357

[b26] Yang H. W. . A small subunit processome protein promotes cancer by altering translation. Oncogene 34, 4471–4481, doi: 10.1038/onc.2014.376 (2015). 25435373

[b27] Yu Y. H. . Network biology of tumor stem-like cells identified a regulatory role of CBX5 in lung cancer. Sci. Rep. 2, 584, doi: 10.1038/srep00584 (2012). 22900142 PMC3419921

[b28] Gerlinger M. . Genomic architecture and evolution of clear cell renal cell carcinomas defined by multiregion sequencing. Nat. Genet. 46, 225–233, doi: 10.1038/ng.2891 (2014). 24487277 PMC4636053

[b29] Sakoda L. C. . Germ line variation in nucleotide excision repair genes and lung cancer risk in smokers. Int. J. Mol. Epidemiol. Genet. 3, 1–17 (2012). 22493747 PMC3316453

[b30] Garcia-Carbonero R., Carnero A. & Paz-Ares L. Inhibition of HSP90 molecular chaperones: moving into the clinic. Lancet Oncol. 14, e358–369, doi: 10.1016/S1470-2045(13)70169-4 (2013). 23896275

[b31] Fusco A. & Fedele M. Roles of HMGA proteins in cancer. Nat. Rev. Cancer 7, 899–910, doi: 10.1038/nrc2271 (2007). 18004397

[b32] Wang D. Z., Ray P. & Boothby M. Interleukin 4-inducible phosphorylation of HMG-I(Y) is inhibited by rapamycin. J. Biol. Chem. 270, 22924–22932 (1995). 7559428 10.1074/jbc.270.39.22924

[b33] Farin K., Di Segni A., Mor A. & Pinkas-Kramarski R. Structure-function analysis of nucleolin and ErbB receptors interactions. PloS One 4, e6128, doi: 10.1371/journal.pone.0006128 (2009). 19578540 PMC2700965

[b34] Humphries J. D. . Proteomic analysis of integrin-associated complexes identifies RCC2 as a dual regulator of Rac1 and Arf6. Sci. Signal. 2, ra51, doi: 10.1126/scisignal.2000396 (2009). 19738201 PMC2857963

[b35] Behrends C., Sowa M. E., Gygi S. P. & Harper J. W. Network organization of the human autophagy system. Nature 466, 68–76, doi: 10.1038/nature09204 (2010). 20562859 PMC2901998

[b36] Asghar U., Witkiewicz A. K., Turner N. C. & Knudsen E. S. The history and future of targeting cyclin-dependent kinases in cancer therapy. Nat. Rev. Drug Discov. 14, 130–146, doi: 10.1038/nrd4504 (2015). 25633797 PMC4480421

[b37] Lurje G. & Lenz H. J. EGFR signaling and drug discovery. Oncology 77, 400–410, doi: 10.1159/000279388 (2009). 20130423

[b38] Gioia R. . Quantitative phosphoproteomics revealed interplay between Syk and Lyn in the resistance to nilotinib in chronic myeloid leukemia cells. Blood 118, 2211–2221, doi: 10.1182/blood-2010-10-313692 (2011). 21730355

[b39] Browne B. C. . Global characterization of signalling networks associated with tamoxifen resistance in breast cancer. FEBS J. 280, 5237–5257, doi: 10.1111/febs.12441 (2013). 23876235

[b40] Martins L. R. . Targeting CK2 overexpression and hyperactivation as a novel therapeutic tool in chronic lymphocytic leukemia. Blood 116, 2724–2731, doi: 10.1182/blood-2010-04-277947 (2010). 20660292

[b41] Kim J. S. . Protein kinase CK2alpha as an unfavorable prognostic marker and novel therapeutic target in acute myeloid leukemia. Clin. Cancer Res. 13, 1019–1028, doi: 10.1158/1078-0432.CCR-06-1602 (2007). 17289898

[b42] Silva A. . PTEN posttranslational inactivation and hyperactivation of the PI3K/Akt pathway sustain primary T cell leukemia viability. J. Clin. Invest. 118, 3762–3774, doi: 10.1172/JCI34616 (2008). 18830414 PMC2556239

[b43] Piazza F. A. . Multiple myeloma cell survival relies on high activity of protein kinase CK2. Blood 108, 1698–1707, doi: 10.1182/blood-2005-11-013672 (2006). 16684960

[b44] Chon H. J., Bae K. J., Lee Y. & Kim J. The casein kinase 2 inhibitor, CX-4945, as an anti-cancer drug in treatment of human hematological malignancies. Front. Pharmacol. 6, 70, doi: 10.3389/fphar.2015.00070 (2015). 25873900 PMC4379896

[b45] Prins R. C. . CX-4945, a selective inhibitor of casein kinase-2 (CK2), exhibits anti-tumor activity in hematologic malignancies including enhanced activity in chronic lymphocytic leukemia when combined with fludarabine and inhibitors of the B-cell receptor pathway. Leukemia 27, 2094–2096, doi: 10.1038/leu.2013.228 (2013). 23900138

[b46] Bliesath J. . Combined inhibition of EGFR and CK2 augments the attenuation of PI3K-Akt-mTOR signaling and the killing of cancer cells. Cancer lett. 322, 113–118, doi: 10.1016/j.canlet.2012.02.032 (2012). 22387988

[b47] Benecke A. G. & Eilebrecht S. RNA-Mediated Regulation of HMGA1 Function. Biomolecules 5, 943–957 (2015). 26117853 10.3390/biom5020943 PMC4496703

[b48] Abe N. . Pancreatic duct cell carcinomas express high levels of high mobility group I(Y) proteins. Cancer Res. 60, 3117–3122 (2000). 10866296

[b49] Balcerczak M. . HMGI(Y) gene expression in colorectal cancer: comparison with some histological typing, grading, and clinical staging. Pathol. Res. Pract. 199, 641–646, doi: 10.1078/0344-0338-00475 (2003). 14666966

[b50] Chiappetta G. . HMGA1 protein overexpression in human breast carcinomas: correlation with ErbB2 expression. Clin. Cancer Res. 10, 7637–7644, doi: 10.1158/1078-0432.CCR-04-0291 (2004). 15569996

[b51] Liau S. S., Ashley S. W. & Whang E. E. Lentivirus-mediated RNA interference of HMGA1 promotes chemosensitivity to gemcitabine in pancreatic adenocarcinoma. J. Gastrointest. Surg. 10, 1254–1262, discussion 1263, doi: 10.1016/j.gassur.2006.06.011 (2006). 17114012

[b52] Belton A. . HMGA1 induces intestinal polyposis in transgenic mice and drives tumor progression and stem cell properties in colon cancer cells. PloS One 7, e30034, doi: 10.1371/journal.pone.0030034 (2012). 22276142 PMC3262796

[b53] Shah S. N. . HMGA1: a master regulator of tumor progression in triple-negative breast cancer cells. PloS One 8, e63419, doi: 10.1371/journal.pone.0063419 (2013). 23658826 PMC3642138

[b54] Zhang Y. . High-mobility group A1 proteins enhance the expression of the oncogenic miR-222 in lung cancer cells. Mol. Cell. Biochem. 357, 363–371, doi: 10.1007/s11010-011-0907-1 (2011). 21656127

[b55] Hillion J. . Upregulation of MMP-2 by HMGA1 promotes transformation in undifferentiated, large-cell lung cancer. Mol. Cancer Res. 7, 1803–1812, doi: 10.1158/1541-7786.MCR-08-0336 (2009). 19903768 PMC3069640

[b56] Han C. L. . A multiplexed quantitative strategy for membrane proteomics: opportunities for mining therapeutic targets for autosomal dominant polycystic kidney disease. Mol. Cell Proteomics 7, 1983–1997, doi: 10.1074/mcp.M800068-MCP200 (2008). 18490355

[b57] Tsai C. F. . Immobilized metal affinity chromatography revisited: pH/acid control toward high selectivity in phosphoproteomics. J. Proteome Res. 7, 4058–4069, doi: 10.1021/pr800364d (2008). 18707149

[b58] Tsai C. F. . Sequential phosphoproteomic enrichment through complementary metal-directed immobilized metal ion affinity chromatography. Anal. Chem. 86, 685–693, doi: 10.1021/ac4031175 (2014). 24313913

[b59] Tsou C. C. . IDEAL-Q, an automated tool for label-free quantitation analysis using an efficient peptide alignment approach and spectral data validation. Mol. Cell Proteomics 9, 131–144, doi: 10.1074/mcp.M900177-MCP200 (2010). 19752006 PMC2808259

[b60] Wang Y. T. . An informatics-assisted label-free quantitation strategy that depicts phosphoproteomic profiles in lung cancer cell invasion. J. Proteome Res. 9, 5582–5597, doi: 10.1021/pr100394u (2010). 20815410

