# Supplementary material for: Phosphoproteomics Reveals HMGA1, a CK2 Substrate, as a Drug-Resistant Target in Non-Small Cell Lung Cancer
Source: Sci Rep. 2017 Mar 14;7:44021. doi: 10.1038/srep44021 (PMC5349541; doi:10.1038/srep44021)
Supplement: Supplementary Information [file srep44021-s1.pdf]

Title: Phosphoproteomics Reveals HMGA1, a CK2 Substrate, as a Drug-Resistant Target in Non-Small Cell Lung Cancer

Yi-Ting Wang , Szu-Hua Pan , Chia-Feng Tsai , Ting-Chun Kuo , Yuan-Ling Hsu, Hsin-Yung Yen,
Wai-Kok Choong , Hsin-Yi Wu , Yen-Chen Liao, Tse-Ming Hong, Ting-Yi Sung, Pan-Chyr Yang and Yu-Ju Chen

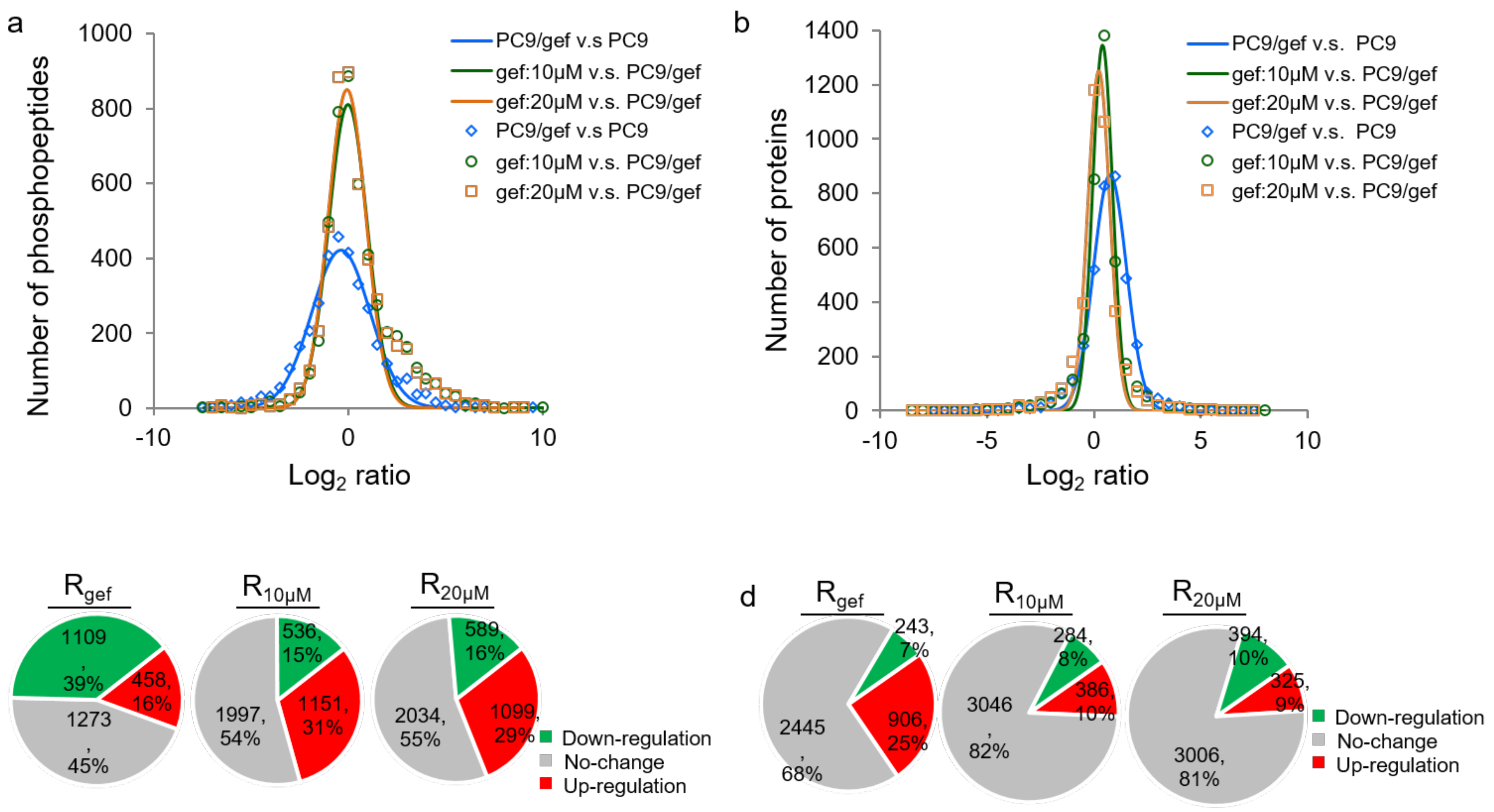

Supplementary Figure S1. **Phosphoproteomics and proteomics analysis of PC9/gef versus PC9, gefitinib 10μM and 20μM treatment of PC9/gef cells compare to PC9/gef.** (**a**) Log_2 ratios distribution for phosphopeptides analysis. (**b**) Log_2 ratios distribution for proteins analysis. (**c**) For phosphoproteomics analysis, pie charts reveal the percentage of 2-fold up-regulation, 2-fold down-regulation and no change of phosphopeptides. (**d**) For proteomics analysis, pie charts reveal the percentage of 2-fold up-regulation, 2-fold down-regulation and no change of proteins.

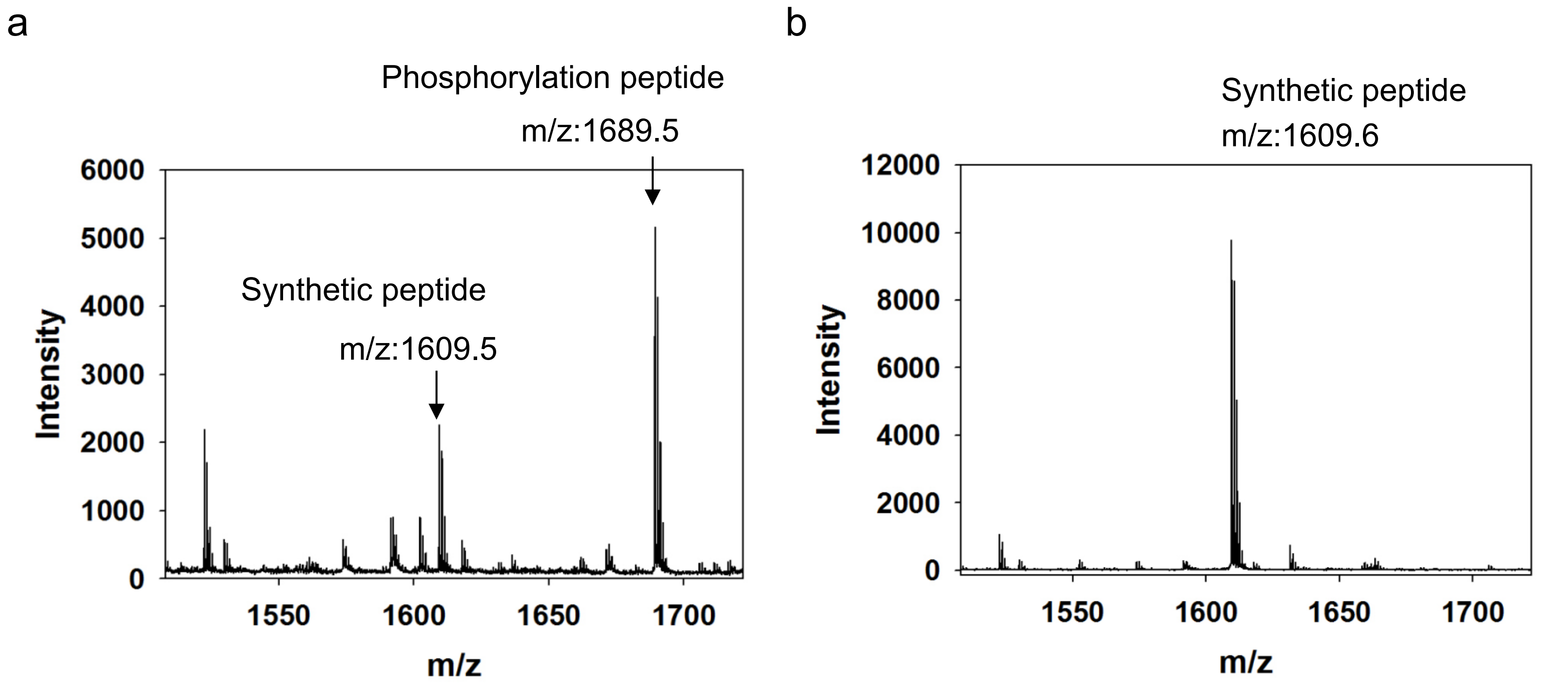

Supplementary Figure S2. *in vitro* kinase assay for synthetic peptide of HMGA1 and detect by MALDI-TOF MS. (**a**) *in vitro* kinase assay with CK2 kinase (**b**). *in vitro* kinase assay with MAP kinase.

a

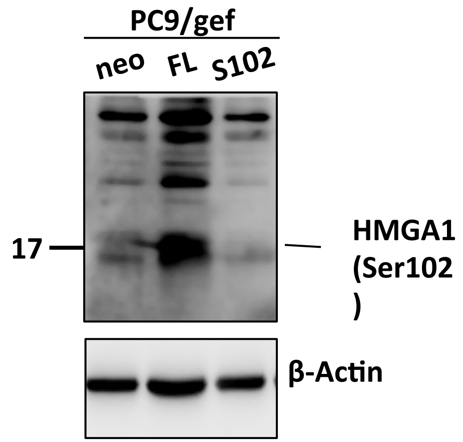

b

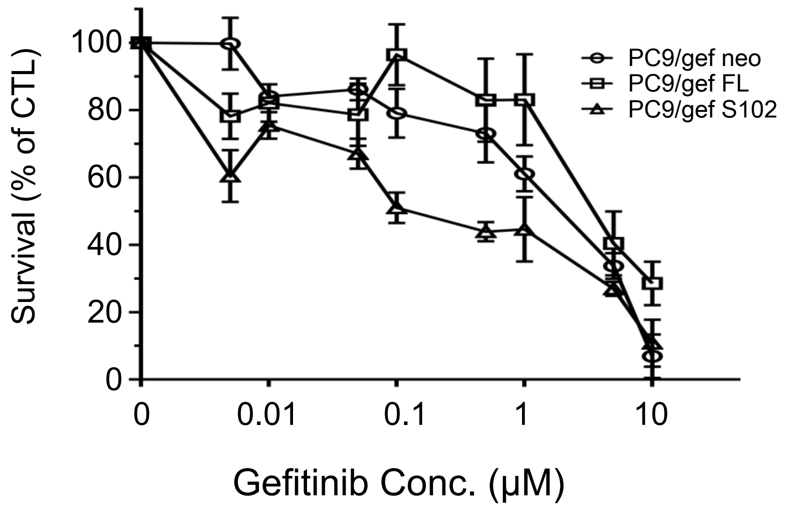

c

Group	neo	FL	S102
IC_{50} (uM)	2.660	3.944	0.197

Supplementary Figure S3. HMGA1 phosphrylated site, S102 regulates drug resistance in NSCLC. (a) the efficiency of phosphorylation site mutation was confirmed by western blot in PC9/gef cells (neo: PC9/gef transfect pCIneo (vector only) plasmid; FL:PC9/gef transfect pCIneo-HMGA1 WT (wild type) plasmid; S102: PC9/gef transfect pCIneo- HMGA1 S102 mutant plasmid), respectively. (b) Cytotoxicity assays for various concentrations of gefitinib in HMGA1 phosphorylation mutation in PC9/gef cells in neo, FL and S102 condition. (c)The IC_{50} of PC9/gef cells treatment with gefitinib in neo, FL and S102 condition.
